# Bread and Meat

**Published:** 1892-02

**Authors:** 


### BREAD AND MEAT.

Said Stiggins to his wife one day,
" We've nothing left to eat;
If things go on in this queer way,
We shan't make *both ends meet.*"

The dame replied in words discreet,
" We're not so badly fed,
If we can make but *one* end *meat*,
And make the other *bread.*"

